# The association between creativity and 7R polymorphism in the dopamine receptor D4 gene (DRD4)

**DOI:** 10.3389/fnhum.2013.00502

**Published:** 2013-08-26

**Authors:** Naama Mayseless, Florina Uzefovsky, Idan Shalev, Richard P. Ebstein, Simone G. Shamay-Tsoory

**Affiliations:** ^1^Department of Psychology, University of HaifaHaifa, Israel; ^2^Department of Psychology, The Hebrew University of JerusalemJerusalem, Israel; ^3^Department of Human Genetics, Hadassah-Hebrew University of JerusalemJerusalem, Israel; ^4^Department of Psychology, National University of SingaporeSingapore

**Keywords:** DRD4, creativity, flexibility, divergent thinking, dopamine

## Abstract

Creativity can be defined as the ability to produce responses that are both novel and appropriate. One way to assess creativity is to measure divergent thinking (DT) abilities that involve generating multiple novel and meaningful responses to open-ended questions. DT abilities have been shown to be associated with dopaminergic (DA) activity, and impaired DT has been reported in populations with DA dysfunctions. Given the strong association between DT and the DA system, the current study examined a group of healthy individuals (*N* = 185) to determine the role of repeat polymorphism in exon3 of the DRD4 gene in creativity. The results show that individuals carrying the DRD4-7R allele scored significantly lower on tests of DT, particularly on the flexibility dimension of DT, compared to non-carriers. The current findings link creative cognition to the DA system and suggest that DA dysfunctions in neurological and psychiatric disorders may account for impaired creativity and cognitive flexibility in these individuals.

## Introduction

Creative cognition plays an important role in the arts, in invention and innovation, as well as in everyday life (Runco and Richards, 1997). Although creativity has been considered a unique human capacity, spontaneously creative behaviors (e.g., creating new tools among primates and birds) have been shown to occur in non-humans as well (Byrne and Bates, 2007), further attesting to creativity’s deep evolutionary and biological roots. Creativity has been defined as the ability to produce responses that are both novel (i.e., original, rare and unexpected) and suitable (i.e., adaptive and useful according to task constraints) (Sternberg and Lubart, 1999). One of the psychometric approaches to measuring creativity involves divergent thinking (DT) tasks in which participants are asked to respond to a given problem with multiple solutions (Dietrich and Kanso, 2010). Tests of DT generally measure various aspects of creativity, including creative fluency, flexibility and originality (Torrance, 1974). Thus, although not synonymous with creativity, DT tasks provide structured and objective measurements of creativity (Sternberg and Lubart, 1999; Jung et al., 2009). Notably, scores on DT tasks have been shown to be positively correlated with ecologically valid measures of creative achievement (Carson et al., 2005) as well as with self-rated creativity (Furnham and Bachtiar, 2008).

Previous studies have pointed to the involvement of the dopaminergic (DA) system in creativity (Heilman et al., 2003; Flaherty, 2005; Takeuchi et al., 2010). Takeuchi et al. (2010) found individual differences in creativity, as measured by DT, to be positively correlated with grey matter in DA system regions, including the dorsolateral prefrontal cortex, bilateral basal ganglia, substantia nigra, and the tegmental ventral area. Additionally, several genetic studies have shown a relationship between DT and dopamine neurotransmission (Reuter et al., 2006; De Manzano et al., 2010; Runco et al., 2011). For example, Reuter et al. (2006) found creativity, as measured by DT tasks involving both figural and verbal creativity, to be significantly associated with polymorphisms of the dopamine D2 receptor gene (DRD2). Moreover, several studies have found cortical dopamine to be involved in cognitive flexibility (Frank, 2005; Cools, 2008; Garcia-Garcia et al., 2010), one of the main components of DT.

One interesting DA candidate gene for creativity is the dopamine D4 receptor gene (DRD4). The DRD4 receptor is one of five dopamine receptors and plays an important role in mediating synaptic dopamine signaling. The gene is characterized by a 48 base-pair variable number of tandem repeats (48-bp VNTR) located in the coding region of the third exon. DRD4 48-bp VNTR polymorphism varies from 2 to 11 repeats across individuals (Asghari et al., 1995; Cravchik and Goldman, 2000), where the 4-repeat (4R) is the most common repeat in Caucasian populations and the 7-repeat (7R) is the second most common variant (Chang et al., 1996; Ding et al., 2002). Interestingly, the 7R has been previously associated with real-life creative behaviors, such as the novelty-seeking personality trait (Ebstein et al., 1996). Indeed, novelty-seeking—the tendency toward exploratory activity—is thought to be one of the characteristics of creative people (Chavez-Eakle et al., 2006; Drago et al., 2009). Although the evidence for an association between DRD4 7R and novelty-seeking is inconsistent, as evidenced by several meta-analysis reports (Kluger et al., 2002; Munafò et al., 2008), many studies have found a significant association between novelty-seeking and the 7R allele of the DRD4 (Ebstein et al., 1996; Benjamin et al., 2000; Keltikangas-Järvinen et al., 2002; Becker et al., 2005). Roussos et al. (2009) have recently suggested that differences in measuring novelty-seeking using self-report scales may account for the inconsistent results between 7R polymorphism and novelty-seeking. In line with this, it has been repeatedly found that the DA system plays a major role in the personality trait of novelty-seeking (Flaherty, 2005; Schweizer, 2006), further attesting to the potential role of DRD4 in creativity.

Nonetheless, in contrast to the role of DRD4 48-bp VNTR 7R in novelty-seeking, recent evidence indicates that 7R is actually associated with impaired cognitive flexibility, one of the major aspects of creativity (Strobel et al., 2004; Congdon et al., 2008). Flexibility involves the ability to make an alternative response after successfully inhibiting a current response. Findings so far on the association between inhibition and DRD4 are mixed. Congdon et al. (2008) found that participants with the 7R allele of the DRD4 exhibit higher stop-signal reaction time (SSRT) on a go/no-go task, reflecting poorer inhibitory control, while Forbes et al. (2009) failed to find such an effect on the Barratt Impulsiveness Scale (BIS), a self-report tool to measure impulsivity. On the other hand, Colzato et al. (2010) demonstrated that these mixed effects may be due to the fact that previous studies addressed impulsivity as a monolithic process, while impulsivity may actually be divided into functional and dysfunctional types according to Dickman’s Impulsivity Inventory (DII; Dickman, 1990). Dysfunctional impulsivity is the tendency to act without forethought in cases when such action is inappropriate, while functional impulsivity is a similar tendency implemented in appropriate situations (Colzato et al., 2010). Colzato et al. (2010) found that individuals with DRD4 7R, which is associated with higher levels of striatal DA, exhibited higher dysfunctional impulsivity. Several meta-analytical studies have pointed to the 7R allele of the DRD4 as a risk allele for attention-deficit hyperactivity disorder (ADHD; Faraone et al., 2001; DiMaio et al., 2003), a disorder characterized by high dysfunctional impulsivity (Young et al., 2007). Furthermore, the relationship between ADHD and creativity has been investigated in several studies (Shaw and Brown, 1990; Healey and Rucklidge, 2006), though so far these studies have yielded unclear results. While some have found ADHD and its symptomology to be positively correlated with creativity (Healey and Rucklidge, 2006; White and Shah, 2011), others have reported an opposite trend (Funk et al., 1993; Healey and Rucklidge, 2005, 2008). Interestingly, ADHD is behaviorally associated with impairments in executive control, including attentional set-shifting (Boonstra et al., 2005), and flexibility (Barkley et al., 1997; Sergeant et al., 2002), both of which are important for cognitive flexibility, a central dimension of creativity (Dietrich, 2004; Durstewitz and Seamans, 2008). These findings indicate that individuals with the 7R allele of the DRD4 may exhibit lower levels of creativity and diminished flexibility on DT tasks in particular. Hence, the DRD4 exon III VNTR is a biologically plausible candidate for contributing to individual differences in creativity.

Collectively, it appears that on the one hand the 7R allele, as a risk allele for impaired executive functions, flexibility and set shifting, may be associated with low creativity. On the other hand, the association between the 7R allele and higher novelty-seeking indicates that individuals with the 7R allele may actually exhibit greater flexibility and creativity.

The current study was designed to explore the complex role of the DRD4 7R in partially shaping human creativity. To better characterize this relationship, we assessed two types of DT tests (figural and verbal). The scoring of each task was divided according to the three main dimensions of DT, namely creative fluency, flexibility and originality, and each dimension was analyzed separately to test the hypothesis that DRD4 differentially affects each dimension of creativity.

## Method

### Participants

The sample comprised 185 students from the University of Haifa in Israel (112 female). All participants were Caucasian (self-reported), with a mean age of 24.5 (SD ± 2.1). Participants were recruited through announcements posted at the university. All participants were paid volunteers.

### Assessment of Creativity

Creativity was assessed using two DT tests, a sub-scale from the figural sub-test of the Torrance test of creative thinking (Torrance, 1974), and the Alternate Uses Task (AUT; Guilford et al., 1978). Both tests included the three core dimensions of DT, namely flexibility, originality and fluency.

### Torrance test of creative thinking (circles sub-scale)

Participants were given a page on which 30 identical circles were drawn. They were asked to draw as many different meaningful objects as possible within a time limit of 10 min, where each drawing must include at least one circle. Scoring included fluency (number of answers produced), flexibility (number of categories) and originality, calculated according to the scoring of original responses, as detailed in the Torrance Tests of Creative Thinking scoring guide (Torrance, 1974).

### Alternate uses task (AUT)

Participants were given a list of five common objects and asked to list as many alternate uses as possible for each object, within a time limit of 10 min. The most common everyday use was indicated in parenthesis. The objects were: shoe (common use: wear on foot); button (common use: closing things); pencil (common use: drawing or writing); tire (common use: car wheel); and drinking glass (common use: contains liquid). Only responses that did not reiterate the given common uses were counted and included. As in the Torrance test, scoring included fluency, flexibility and originality. Since there are no guidelines for the scoring of original responses in the AUT, original responses were defined as statistically infrequent responses according to a pretest conducted in our lab.

### Pretest

For the purpose of creating a valid criterion of response frequency, a group of 100 healthy participants who did not take part in this study completed the AUT. For each object, a list of all possible uses was collected from all participants. A statistical infrequency measure was calculated based on this list in order to evaluate the originality score for each answer, and, subsequently, for each participant. Answers were assigned a score of zero if 5% or more of the participants listed a given use, a score of one if between 2% and 4.99% of participants listed it, and a score of two if less than 1.99% listed the use. An average originality score was calculated for each participant according to these statistical infrequency scores.

#### DNA extraction and genotyping

DNA was extracted from 20 ml of mouthwash samples using the Master Pure kit (Epicentre, Madison, WI). The DRD4 48-bp VNTR was characterized by a PCR amplification procedure with the following primers: F5′ - CTT CCT ACC CTG CCC GCT CAT GCT GCTGCT CTA CTGG - 3′ and R5′ - ACC ACCACC GGC AGG ACC CTC ATG GCC TTG CGC TC – 3. PCR reactions were conducted using 5 µl Master Mix (Thermo scientific), 2 µl primers (0.5 µM), 0.6 µl Mg/Cl2 (2.5 mM), 0.4 µl DMSO 5% and 1 µl of water to a total of 9 µl total volume, and an additional 1 µl of genomic DNA was added to the mixture. All PCR reactions were carried out on a Biometra T1 Thermocycler (Biometra, Güttingem, Germany). The PCR reaction condition was as follows: preheating step at 94.0°C for 5 min, 34 cycles of denaturation at 94.0°C for 30 s, reannealing at 55°C for 30 s, and extension at 72°C for 90 s. The reaction proceeded to hold at 72°C for 5 min. The reaction mixture was then electrophoresed on a 3% agarose gel (AMRESCO) with ethidium bromide to screen for genotypes.

The distribution of genotype frequency was according to the Hardy-Weinberg equilibrium (chi-square = 0.01, *p* value = 0.92).

### Statistical analysis

Genotype was classified according to the presence or absence of the 7R allele of the DRD4 (7 vs. no7), as in line with previous reports in the literature (Ding et al., 2002). Group differences on creativity scores were analyzed statistically using ANOVAs, with the presence (7) or absence (no7) of the DRD4 7R allele as the independent variable. To examine the different aspects of DT (fluency, flexibility, originality), we conducted a multivariate analysis of variance (MANOVA) separately for each component.

## Results

To confirm that the two groups (7, no7) were not significantly different in terms of age and education, we conducted independent t-tests to compare the two groups (see Table 1 for means and standard deviations). This analysis revealed that the two groups did not differ in terms of age or years of education. Non-parametric tests (Mann–Whitney) revealed significant differences in the frequency distribution for gender (*Z* = 2.11, *p* < 0.05).

**Table 1:** **Age, gender and education data for the DRD4 7R genotype, SD: standard deviation**.

****	**N**	**Female (percent)**	**Age (years)**		**Education (years)**	
****	****	****	**Mean**	**SD**	**Mean**	**SD**
no7	128	65.63	24.54	2.2	13.83	1.27
7	57	49.12	24.67	2.02	13.82	1.16

The mean scores of the creativity measures used in the two tasks are summarized in Table 2.

**Table 2:** **Means and Standard Error (SE) of the two creativity tasks used: AUT and Torrance**.

****	****	**7**		**no7**	
****	****	**Mean**	**SE**	**Mean**	**SE**
Fluency	Torrance	12.77	0.69	14.35	0.53
	AUT	3.72	0.17	4.24	0.13
Flexibility	Torrance	8.74	0.39	10.39	0.38
	AUT	3.36	0.15	3.78	0.11
Originalty	Torrance	27.37	1.28	31.07	1.10
	AUT	2.50	0.20	2.83	0.17

### The fluency component

**Figure 1:**
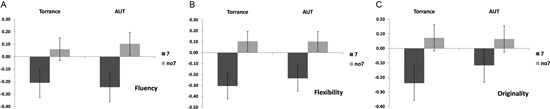
**Creativity scores on the AUT and Torrance tasks separated to fluency (A), flexibility (B) and originality (C)**. Means were transformed into Z scores.

A multivariate ANOVA indicated a general effect of DRD4 7R genotype for the fluency component, *F*(2,182) = 3.25, *p* < 0.05. Given the significance of the overall test, univariate main effects were examined. Significant univariate main effects for DRD4 genotype were obtained for AUT fluency *F*(1,183) = 4.95, *p* < 0.05 (7R allele exhibiting lower fluency compared to the no7 group, Figure 1A), but did not reach the level of significance for Torrance fluency *F*(1,183) = 2.95, *p* = 0.087.

### The flexibility component

Multivariate ANOVA of the flexibility measures exhibited a general effect for DRD4 7R genotype *F*(2,182) = 4.38, *p* < 0.05. Significant univariate main effects for DRD4 genotype were obtained for AUT flexibility *F*(1,183) = 4.57, *p* < 0.05 and for Torrance flexibility *F*(1,183) = 6.95, *p* < 0.01. As shown in Figure 1B, in both cases individuals in the 7R allele group exhibited lower flexibility compared to individuals in the no7 group (Table 2).

### The originality component

Multivariate ANOVA of the originality measures did not exhibit any significance effects, *F*(2,182) = 2.06, *p* = 0.13, although the scores did indicate lower originality for the 7R allele of the DRD4 (see Figure 1C, Table 2 for details).

### Gender differences

Because gender distribution differed significantly between the two DRD4 7R groups, we re-analyzed the multivariate ANOVA’s, with gender as an additional independent between-subject variable. These analyses did not indicate any interaction effects for gender and DRD4 genotype, either for fluency (*F*(2,180) = 0.81, *p* = 0.45) or for flexibility (*F*(2,180) = 1.55, *p* = 0.21). Adding gender as an independent variable rendered the main effect of DRD4 genotype on fluency as not significant, *F*(2,180) = 2.73, *p* = 0.068, but did not change the significance of the effect for flexibility, *F*(2,180) = 3.39, *p* < 0.05. There were no significant main effects for gender either on fluency (*F*(2,180) = 2.79, *p* = 0.064) or on flexibility (*F*(2,180) = 1.63, *p* = 0.19).

## Discussion

DT tasks require participants to provide multiple solutions to a given problem. In the tasks used here, participants were required to think of many alternate possible uses for everyday items (in the AUT), and to draw multiple drawings incorporating a specific shape (circle) (in the Torrance task). The results presented here demonstrate that individuals with the DRD4 7R allele exhibit lower creativity as measured by DT. Considering that the 7R allele has been associated with dysfunctional impulsivity and poor inhibition (Congdon et al., 2008; Dreber et al., 2009), the fact that individuals with the 7R allele exhibit lower creativity can be explained by their inability to suppress or inhibit obvious (common) responses, as indicated by lower originality scores. Lower flexibility and fluency scores may indicate that once a common response has been given, it becomes harder to shift to a new category (as indicated by the low flexibility scores), leading to fewer responses (as indicated by the low fluency score). Further evidence for the relationship between creativity and impulsivity can be seen in the association of the 7R allele with ADHD (Li et al., 2006), as well as with increased impulsivity in ADHD patients (Langley et al., 2004). Interestingly, creativity has been linked to the DA system in the case of schizophrenia (Eysenck, 1993). Although several studies show that patients with schizophrenia and schizotypal personality exhibit high creativity (O’Reilly et al., 2001; Folley and Park, 2005), recent models of schizophrenia and creativity support an inverted U-shaped association in which creativity may be higher with low-moderate schizotypal trait, but decreases as the severity of psychopathology in schizophrenia increases (Tsakanikos and Claridge, 2005; Stoneham and Coughtrey, 2009; Nelson and Rawlings, 2010). Indeed, in a recent study Jaracz et al. (2012) reported that patients with schizophrenia show diminished creativity compared to controls. Furthermore, the authors reported that impaired creativity was associated with low cognitive flexibility and impulsivity, reflected by their low scores on the Wisconsin Card Sorting Test (WCST). These results are in line with previous research showing that patients with chronic schizophrenia exhibit lower creativity, which in the case of creative fluency was mediated by scores on executive control tasks and in the case of originality was not (Abraham et al., 2007). Collectively, while mild functional impulsivity may improve creativity among patients with schizotypal traits, especially in generating ideas which differ from the examples given (Abraham and Windmann, 2008), the dysfunctional impulsivity observed in severe schizophrenia may dampen creative abilities.

The results of the current study support the relationship between creativity and variants of DA genetic polymorphisms (Reuter et al., 2006; Runco et al., 2011). Moreover, the results found in our sample indicate that the main effect of the DRD4-7R allele on creativity may be due to its impact on flexibility. Several studies have linked cognitive flexibility with DA pathways (Ashby and Isen, 1999; Dreisbach and Goschke, 2004; Müller et al., 2007). It has been postulated that activation of D2-like receptors (i.e., D2, D3 and D4) decreases GABAergic inhibition and facilitates activation of multiple representations, thus increasing flexibility as manifested by set-shifting abilities (Müller et al., 2007; Seamans and Robbins, 2010). Moreover, D2-like receptors have been linked to flexible integration of new information (Durstewitz and Seamans, 2008). In line with this, Reuter et al. (2006) found an association between the dopamine receptor D2 and creativity as measured by a composite index, though whether flexibility played a major role in these results cannot be determined. Additionally, Runco et al. (2011) reported differences in creative fluency among carriers of several dopamine genes, among them the DRD4. However, it is not clear from their report which group exhibits greater creative abilities. It is important to note that as opposed to the results presented here, Runco et al. (2011) did not find an association between measures of flexibility as measured by DT tasks and DRD4. This inconsistency may be explained by differences in the populations used and the group sizes or by differences in allele frequencies within the sampled population. Furthermore, there is evidence suggesting that the association between dopamine and DT may not be a linear one (Akbari Chermahini and Hommel, 2010, 2012), indicating that an interaction among several DA genes may influence the individual results of each genetic variability in the context of DT. In addition, several studies have pointed to the fact that DA pathways may be gender sensitive (Tammimäki and Männistö, 2011; Wang et al., 2012). Zhong et al. (2010), for example, found that gender modulated the association between DRD4 exon3 polymorphism and sense of fairness. In the current study we did not find an interaction effect between the DRD4-7R and gender, indicating that gender does not have a modulating effect on the association between DRD4 and creativity in our sample. Furthermore, the results presented here indicate a lack of gender differences in creativity, and in DT in particular (for review, see Baer and Kaufman, 2008).

In conclusion, in the current study we suggest that the association found between DRD4 and creativity is mainly influenced by flexibility. The results of the current study are in line with previous results pointing to the involvement of the DA system in creativity and add to the accumulating body of knowledge suggesting genetic influences in creativity.

There are several limitations of the study that should be acknowledged. First, the current study only examined the association between one genetic variability in the DA system—the DRD4—and DT. However, there are other important polymorphisms in the striatal DA system that could be associated with DT and may contribute both in a standalone manner as well as in gene-gene interactions. One such polymorphism is the dopamine transporter (DAT1), which has been shown to be associated with inhibition of return (Colzato et al., 2010) as well as with measures of impulsivity (Gizer and Waldman, 2012). Furthermore, as mentioned above, another possibility that was not explored here is the possible gene-gene interaction effects on DT. Akbari Chermahini and Hommel (2010) reported a nonlinear association between a marker of striatal dopamine and DT, indicating that the relation may be more complex, such that one polymorphism of a gene may impact DT through the mediation of another gene polymorphism. Thus, future studies should consider examining the interaction between these two polymorphisms in the context of flexibility and DT.

## Conflict of interest statement

The authors declare that the research was conducted in the absence of any commercial or financial relationships that could be construed as a potential conflict of interest.
